# The clinical manifestation and diagnostic features of Kawasaki-like phenotypes in pediatric multisystem inflammatory syndrome: a comparative retrospective study in Ukraine

**DOI:** 10.3389/fped.2025.1593190

**Published:** 2025-07-03

**Authors:** Nataliia Bodnarchuk-Sokhatska, Halyna Pavlyshyn, Kateryna Kozak, Iryna Avramenko

**Affiliations:** ^1^ Department of Pediatrics №2, I. Horbachevsky Ternopil National Medical University, Ternopil, Ukraine; ^2^ Department of Propaedeutics of Pediatrics, Danylo Halytsky Lviv National Medical University, Lviv, Ukraine

**Keywords:** multisystem inflammatory syndrome in children, Kawasaki disease, SARS-CoV-2, phenotypes, clinical characteristics

## Abstract

**Background:**

The clinical overlap syndrome between multisystem inflammatory syndrome in children (MIS-C) and Kawasaki disease (KD), particularly in the context of SARS-CoV-2 infection, presents diagnostic challenges. The presence of both complete and incomplete Kawasaki-like phenotypes (KLP) further complicates differentiation. This study aimed to analyze Kawasaki-like phenotype of MIS-C, its clinical features, and improve diagnostic accuracy, patient outcomes.

**Methods:**

A retrospective cohort study was conducted on 48 pediatric patients diagnosed with MIS-С between 2020 and 2022. All cases met the MIS-C diagnostic criteria established by the Council of State and Territorial Epidemiologists (2022) and were classified according to the American Heart Association Kawasaki disease criteria (2017). Patients were grouped as non–Kawasaki-like or Kawasaki-like MIS-C phenotypes, with the latter subdivided into complete and incomplete subtypes. Clinical and echocardiographic features were compared using appropriate statistical methods.

**Results:**

Among the 48 MIS-C cases analyzed, 22 patients (46%) met the Kawasaki disease criteria, equally divided between complete and incomplete Kawasaki-like phenotypes. btion was longest in the complete phenotype (9.7 days) and shortest in the incomplete phenotype (5.5 days). Patients with neurological involvement experienced longer febrile periods (8.3 vs. 5.4 days). All 100% patients with the complete phenotype exhibited neurological symptoms vs. 46% of incomplete cases. Half of the Kawasaki-like phenotype patients demonstrated echocardiographic abnormalities vs. 15% of non–Kawasaki-like (NKL); highest in the incomplete phenotype (91%) compared to 15% in non–Kawasaki-like and 9% in complete KLP. The highest incidence of coronary dilatation was recorded in the incomplete phenotype (73%) vs. 9% in the complete and 15% in the non–Kawasaki-like MIS-C.

**Discussion:**

Kawasaki-like MIS-C phenotypes display distinct clinical and cardiovascular profiles. Accurate phenotypic identification is crucial for risk stratification and optimizing patient management. Further research is necessary to refine classification criteria and establish effective long-term monitoring strategies for affected children.

## 1 Introduction

The relationship between Kawasaki disease and multisystem inflammatory syndrome in children (MIS-C) stems from their overlapping clinical and laboratory features, making differential diagnosis a significant challenge for physicians across various specialties. The presence of shared clinical signs gives rise to the phenomenon of mimicry (1–3), further complicating accurate identification. This challenge is heightened by the existence of both complete and incomplete forms of Kawasaki disease, which require careful clinical assessment to distinguish them from MIS-C and ensure appropriate management. Verification of Kawasaki-like phenotypes within MIS-C cases, through the integration of diagnostic criteria, enables differentiation between these overlapping conditions. A phenotype-oriented approach to patient management in emergency settings, along with the refinement of prognostic factors based on clinical data are undeniable advantages in building a rapid and accurate diagnostic system for these nosologies. The aim of this study was to analyze and identify the clinical pecularities of MIS-C in children in the observation cohort.

## 2 Materials and methods

### 2.1 Study design

Clinical data for the analytical database were collected from 48 inpatient charts of patients who were treated in healthcare facilities between 2020 and 2022. The study included children under 18 years of age diagnosed with MIS-C, selected based on the criteria established by the Council of State and Territorial Epidemiologists 2022 (4). Phenotypic classification followed the Kawasaki disease criteria outlined by the American Heart Association 2017 (5–7), incorporating the 2024 update. Сhanges in coronary arteries were recorded according to guidelines and standardized echocardioscopy techniques (8, 9).

The patient cohort was categorized based on the presence or absence of diagnostic criteria for Kawasaki disease in the structure of the MIS-C case. Two primary phenotypes were identified: the non-Kawasaki-like and Kawasaki-like phenotypes. The KLP group was further subdivided into two types: complete and incomplete. Statistical analysis at the first stage compared the significance between two phenotypes: non–Kawasaki-like and Kawasaki-like MIS-C, subsequently, multiple statistical analysis was applied between the three formed groups (non–Kawasaki-like, complete and incomplete subtypes Kawasaki-like). Patients who did not meet the criteria of Kawasaki disease were included in the group with the NKL phenotype.

Exclusion criteria. Patients who did not initially meet the MIS-C criteria according to the Council of State and Territorial Epidemiologists 2022 or who exclusively met the definition of Kawasaki disease were not included in the study. Patients with a confirmed other specific etiological diagnosis were also not included in the analysis.

### 2.2 Statistical analysis

Descriptive analyses was performed using the statistical package “Statistical software EZR v. 1.54”. The variables are presented as mean ± SD or median and interquartile range (IQR)—[Me (Q1; Q3)] according to the normal distribution of the data as determined by the Shapiro–Wilk test (W-test). In turn, based on this, further analysis was conducted according to the corresponding criteria: for quantitative traits, two independent groups—parametric (Student's t-test) and non-parametric (Mann–Whitney), multiple (more than 2 groups) comparisons, respectively—ANOVA test and Kruskal–Wallis test. *post-hoc* comparisons were conducted using the Bonferroni correction. For qualitative, categorical, paired variables, Fisher's criterion was used, and considering the sample size, Fisher's exact criterion for more than 2 groups. The threshold for statistical significance was set at *p* = 0.05.

## 3 Results

### 3.1 Phenotypic distribution and demographic features of MIS-C cases

An analysis of 48 clinical cases of MIS-C was conducted. Among them, 22 (46%) patients met the criteria for Kawasaki disease, also, from this group 11 (50%) referred to the complete and incomplete types. Patients who did not meet the criteria for Kawasaki disease were classified into the non–Kawasaki-like MIS-C group—26 (54%). To provide a comprehensive overview of clinical and demographic features across MIS-C phenotypes, like age, sex, and affected organ systems, which is presented in Table 1.

**Table 1:** Comparative analysis of demographic and syndromic patterns in MIS-C cases stratified by phenotype.

Sign		Non–Kawasaki-like phenotype of MIS-C (*n* = 26)	Kawasaki-like phenotype of MIS-C		
			General (*n* = 22)	Complete subtype (*n* = 11)	Incomplete subtype (*n* = 11)
Age, year		6 (3; 10)	7 (3; 10)	10 (3; 10,5)	6 (3,5; 8,0)
*p*-value		0.926			
		0.752			
Gender,	Male	16 (60%)	17 (76%)	7 (60%)	9 (87%)
	Famale	10 (40%)	5 (24%)	4 (40%)	2 (13%)
*p*-value		0.695			
		0.752			
Temperature duration, days		7.4 ± 3.8	7.6 ± 3.9	9.7 ± 3.6	5.5 ± 3.2
*p*-value		0.857			
		0.030			
Mucocutaneous syndrome (*n* = 46)		24 (92%)	22 (100%)	11 (100%)	11 (100%)
*p*-value		0.432			
		0.999			
Gastrointestinal tract (*n* = 32)		20 (77%)	12 (55%)	7 (64%)	5 (46%)
*p*-value		0.134			
		0.213			
Respiratory system (*n* = 32)		16 (62%)	16 (73%)	6 (55%)	10 (91%)
*p*-value		0.543			
		0.132			
Neurological symptoms (*n* = 35)		19 (73%)	16 (73%)	11 (100%)	5 (46%)
*p*-value		0.999			
		0.014			
Urinary system (*n* = 11)		8 (31%)	3 (14%)	1 (9%)	2 (18%)
*p*-value		0.191			
		0.430			

**Figure 1:**
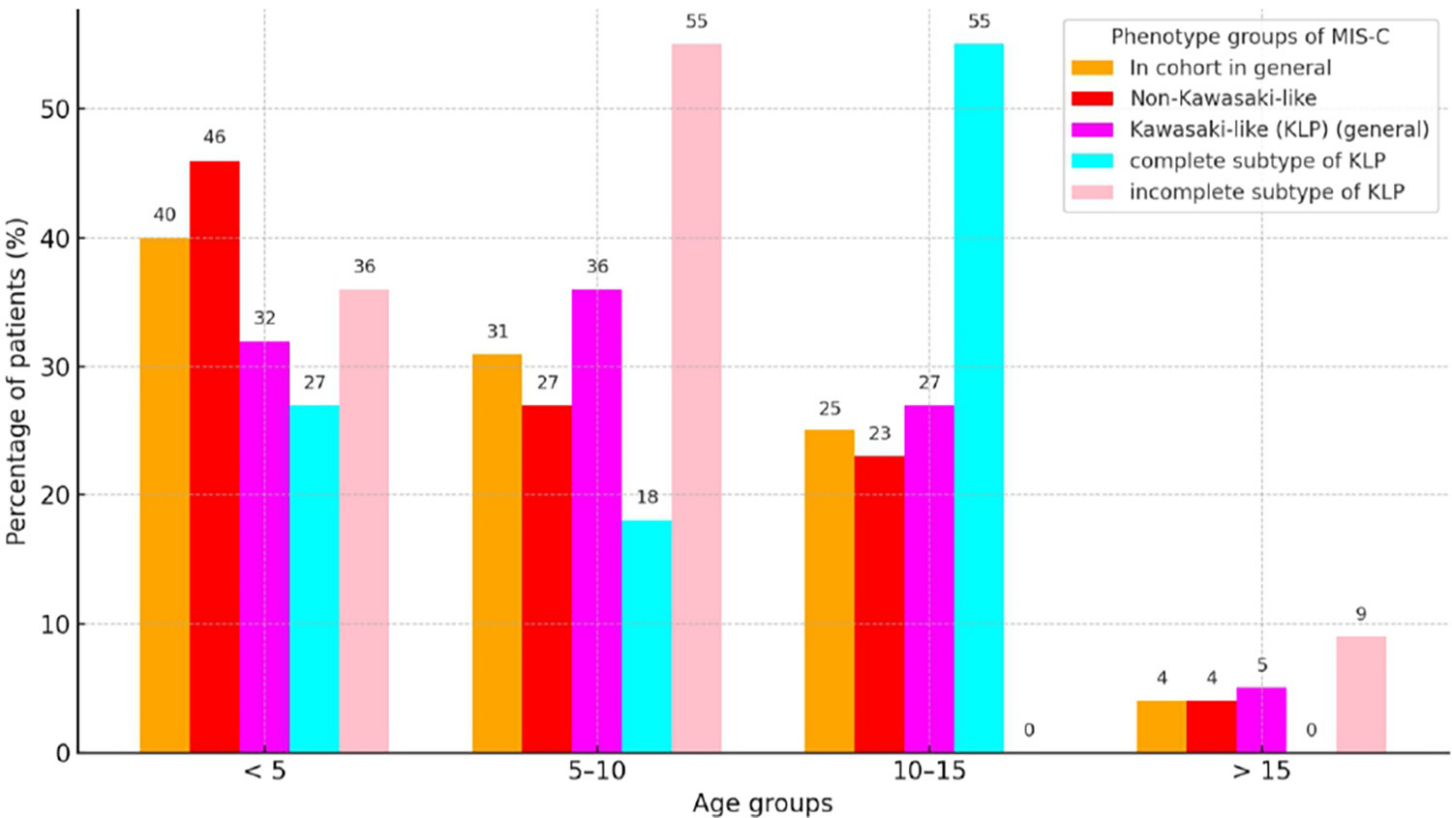
Percentage distribution of age groups across clinical phenotypes of MIS-C cases.

The age of the patients did not show a significant difference between the groups; however, it should be noted that, in clinical terms, the children in the complete KLP were older (median age 10) than incomplete one. The age structure by phenotypic characteristic is graphically visualized in Figure 1. The obtained analysis revealed a certain affinity (the phenomenon of mimicry) between Kawasaki disease and MIS-C, as in general for the patient cohort and in the non–Kawasaki-like phenotype. The largest proportion of MIS-C cases was among children under 5 years old (40% and 46% respectively) with a gradual decrease in proportion in the age category. The fewest patients were observed in all groups in the age category from 15 years old. Notably, there was a predominance of children aged 5–10 and 10–15 years in the group with incomplete and complete KLP (55% each).

In the overall patient group, as well as in the non–Kawasaki-like MIS-C and complete Kawasaki-like phenotype, there was an even gender distribution of 40%. However, in the incomplete subtype, the proportion of girls was significantly lower at 13%. The male-to-female ratio among patients not meeting the criteria for Kawasaki disease was 1.2:1 consistent with overall in the group, while in Kawasaki-like phenotype 1.8:1. Specifically, the complete KLP had a ratio of 1.2:1, whereas the incomplete subtype showed a more pronounced male predominance at 2.6:1.

### 3.2 Hospitalization parameters and comorbidity among MIS-C phenotypes

The duration of the patient's stay in the healthcare facility for children with non–Kawasaki-like MIS-C was 12.5 (11; 16) days and with KLP 10.5 (9; 13.8) days (*p* > 0.05), while the severity of their condition required treatment in the intensive care unit for 13 (50%) and in the second group for 9 (41%) patients, with an average duration of their stay being 3.6 ± 6.2 and 1.7 ± 2.4 days (*p* > 0.05). Children with the complete 5 (46%) and incomplete—4 (36%) subtypes of KLP required intensive care treatment.

Comorbidity was observed in 7 (27%) and 9 (41%) children in the non-Kawasaki-like and Kawasaki-like MIS groups, respectively (*p* > 0.05). Moreover, among analyzed comorbidity, the highest percentage was observed in the incomplete KLP, namely in 5 (46%).

### 3.3 Fever duration and association with clinical manifestation

The duration of fever in children with multisystem inflammatory syndrome averaged as 7.5 ± 3.9 days. A statistically significant difference was found in the multiple comparison (post-hoc level) between both types of KLP [*p* = 0.857, t = −2.9 95% CI (−7.3 to −1.2)]. The analysis between fever duration and specific system involvement revealed a statistical significance with neurological symptoms (*p* = 0.020, t = −2.40). Patients with pathological symptoms from the nervous system had a longer febrile period of 8.3 ± 4.0 compared to 5.4 ± 2.4 days [95% CI (−5.28 to −0.47)].

### 3.4 Systemic involvement across phenotypes

An analysis of organ system involvement, according to Table 1 across MIS-C phenotypes revealed that skin and mucosal lesions occurred in 100% of patients in all groups (*p* > 0.05). Respiratory disorders were most commonly clinically observed in patients with the incomplete Kawasaki-like phenotype (91%), whereas symptoms related to the urinary system (31%) and gastrointestinal tract (77%) predominated among patients with NKL MIS-C.

Neurological symptoms were statistically less frequent, in the group with the incomplete type of MIS-C (46%) compared to the complete type (100%) (*p* = 0.037) [95% CI (0.25–0.84)].

### 3.5 Cardiovascular involvement and phenotype-specific patterns

The characteristics of cardiovascular system damage indicated the presence of changes in the myocardium, pericardium, and coronary vessels highlighting the potential severity of the cardiovascular involvement in MIS-C (Table 2).

**Table 2:** Features of cardiovascular manifestations in patients with different MIS-C phenotypes.

Sign of CVS damage	Non–Kawasaki-like phenotype of MIS-C (*n* = 26)	Kawasaki-like phenotype of MIS-C		
		General (*n* = 22)	Complete subtype (*n* = 11)	Incomplete subtype (*n* = 11)
Hypotension or shock	2 (8%)	2 (9%)	1 (9%)	1 (9%)
	*р* = 0.999			
	*р* = 0.999			
Myocarditis	7 (28%)	3 (14%)	0 (0)	3 (27%)
	*р* = 0.292			
	*р* = 0.172			
Pericarditis	10 (39%)	6 (27%)	3 (27%)	3 (27%)
	*р* = 0.540			
	*р* = 0.771			
Coronary arteritis	1 (4%)	3 (14%)	0 (0)	3 (27%)
	*р* = 0.321			
	*р* = 0.082			
CV dilatation	4 (15%)	9 (41%)	1 (9%)	8 (73%)
	*р* = 0.058			
	*p* < 0.001			
CV aneurysms	0 (0)	2 (9%)	0 (0)	2 (18%)
	*p* = 0.213			
	*p* = 0.097			
Overall prevalence of CVS lesions	17 (65%)	13 (59%)	3 (27%)	10 (91%)
	*р* = 0.999			
	*р* = 0.043			

СV, coronary vessels; CVS, cardiovascular system.

Eight patients (17%) demonstrated a reduced left ventricular ejection fraction (less than 55%). At the same time, half of them were children, with the non–Kawasaki-like phenotype 4 (15%) and the incomplete KLP MIS-C—4 (36%) (*p* = 0.058).

When analyzing the characteristics of structural changes in the heart's blood supply system and their connection with other organ and system lesions during an episode of multisystem inflammatory syndrome, the following features were identified. Posterior pairwise comparisons with Bonferroni correction did not reveal differences between groups. However, clinically, the prevalence of coronaryitis is more frequently observed in the group with the incomplete KLP (27%). It is worth noting the clinical difference in the dilation index in coronary vessels between the non–Kawasaki-like and Kawasaki-like phenotypes of MIS-C, 15% vs. 41%, despite the absence of statistical significance (*p* = 0.058). A statistical difference was found using Fisher's exact test for three independent samples of patients. *post-hoc* comparisons with Bonferroni correction revealed a significant difference between the group with complete and incomplete subtypes of KLP {*p* = 0.004; [95% CI (−0.95 to −0.32)]}, and between non–Kawasaki-like MIS-C and incomplete KLP MIS-C {*p* = 0.021 [95% CI (−0.87 to −0.28)]}. However when checking at the *post-hoc* stage, no differences between groups were found (*p* > 0.05). Also, it should be noted that, clinically, in the case of the incomplete sutype of KLP, cardiovascular system involvement is most frequently observed (82%) compared to other groups.

### 3.6 Echocardiographic and coronary abnormalities

Additionally, the echocardiographic findings among MIS-C patients were analyzed (Table 3). Coronary vessel changes аmong the entire cohort of patients occurred in 15 (31%) cases.

**Table 3:** Relationship of echo-ECG changes in patients with MIS-C with different phenotypes.

Phenotype group	Non–Kawasaki-like phenotype of MIS-C (*n* = 26)	Kawasaki-like phenotype of MIS-C		
		General (*n* = 22)	Complete subtype (*n* = 11)	Incomplete subtype (*n* = 11)
Registered echo-ECG changes in the group, *n* (%)	4 (15)	11 (50)	1 (9)	10 (91)
*p*-value	0.014			
	<0.001			

According to data presented in Table 3, a statistically significant difference was found in the groups of children with both (non- and Kawasaki-like) phenotypes of MIS (*p* = 0.014, OR = 5.3 [95% CI (1.2–28.3)]. Accordingly, the chances of registering echocardioscopic changes in children with Kawasaki-like syndrome are higher than in the NKL phenotype. The analysis of the difference between groups using the independent samples method (Fisher's exact test) confirmed a statistically significant difference (*p* < 0.001). At the *post hoc* stage, pairwise comparisons (with Bonferroni correction) revealed a difference between non–Kawasaki-like MIS-C and incomplete KLP {*p* < 0.0001, [95% CI (−0.98 to −0.54)]}, and between complete and incomplete KD {*p* < 0.001, [95% CI (−1.1 to −0.58)]}. These results indicate a higher likelihood of detecting echocardiographic changes in KLP overall, specifically in its incomplete subtype.

## 4 Discussion

Multisystem inflammatory syndrome in children (MIS-C) exhibits considerable heterogeneity in clinical presentation, and the need for phenotype-specific interpretation has become increasingly recognized. The rarity of cases—ranging from 0.2–11.4 (up to 15 in Japan (10) per 100,000 population (11–13)—necessitates careful differential diagnosis (14, 15).

Percentage distribution in the age and gender aspects of our MIS-C cases, during comparing with CDC data (16) and reported literature (13, 17), followed the same pattern, which confirms the consistency of our patient's cohort with broader epidemiological trends and reinforces the generalizability of the results. The age-related distribution revealed an inverse pattern: children who did not meet the criteria for Kawasaki disease were predominantly under the age of 5, whereas those who did were mostly older than 5 years (18).

In most cases, the distribution of patients into phenotypes in the literature is described as MIS-C with or without shock, Kawasaki-like, or with fever dominance, hyperinflammatory syndrome (19–22), or a specific clinical picture (23). In our study, involvement of the gastrointestinal and urinary systems, predominantly, was characteristic of non–Kawasaki-like phenotype. These findings are consistent with litreture observations (24), who report that gastrointestinal involvement was reported in 87% of cases.

In case of subtypes of Kawasaki-like phenotype central nervous system involvement, higher period of fever and older age was revealed statisticaly. This finding has resembled with literature data (25), which describe the combination of a prolonged febrile period and the manifestation of neurological symptoms in children with MIS-C.

Recent data indicate that patients with incomplete forms of Kawasaki disease may exhibit comparable or even higher rates of cardiovascular involvement than those with complete presentations (26). These findings are consistent with our results, where the incomplete Kawasaki-like phenotype of MIS-C demonstrated the highest frequency of cardiac manifestations. Phenotype-driven predisposition to the development of coronary vessel lesions requires careful clinical and laboratory monitoring to verify subclinical and latent pathological conditions in such patients (27–30).

The actual, statistically significant, predominance of neurological symptoms in the complete subtype of KLP and of cardiovascular manifestations with echocardiographic changes in the incomplete subtype represents critical components in the diagnostic and differential framework for establishing the MIS-C phenotype.

The distinction between the complete and incomplete subtype of Kawasaki-like phenotype in the literature is highlighted by a limited number of publications. At the same time, it should be noted that in studies involving more than a thousand patients, the prevalence of the incomplete type of Kawasaki disease during the coronavirus pandemic has been observed (22). Such trends may explain the overlap syndrome of MIS-C and Kawasaki disease in the context of clinical representation.

The severity of patients' conditions, the high mortality risk, potential for multiple organ complications, and the substantial material and technical demands on healthcare facilities further effective management. Considering recent findings such as those reported (31) which highlight variations in treatment response among MIS-C patients, the importance of clear clinical differentiation based on phenotypic profiles becomes evident. Phenotype-specific stratification may offer improved guidance for tailored therapeutic approaches, particularly in identifying patients who may benefit from adjunctive or intensified treatment modalities.

## 5 Conclusions

The study highlights clinically significant distinctions across MIS-C phenotypes. The non–Kawasaki-like MIS-C phenotype demonstrated more frequent involvement of gastrointestinal and urinary systems, as well as the youngest median age (under 5 years) among the cohorts. Pathological findings in coronary vessels were less commonly observed in this group compared to the Kawasaki-like phenotypes.

Among children who met the criteria for complete Kawasaki disease, the majority were aged 10–15 years. In this subtype, neurological symptoms were present in all cases, while kidney and cardiovascular involvement was less frequent, with no observed cases of myocarditis, coronaryitis, or aneurysms.

In constrant, patients with incomplete KLP, mostly affecting children aged 5–10 years, was statistically associated with the highest rate of coronary abnormalities and echocardiographic changes, alongside shorter fever duration.

Identifying MIS-C phenotypes associated with SARS-CoV-2, based on their mimicry of Kawasaki disease provides a framework for prognostic assessment, enabling phenotype-specific strategies for diagnosis, monitoring to assess potential long-term consequences, and management of such patients.

## Data Availability

The original contributions presented in the study are included in the article/Supplementary Material, further inquiries can be directed to the corresponding author/s.
